# Cancer Health Literacy and Willingness to Participate in Cancer Research and Donate Bio-Specimens

**DOI:** 10.3390/ijerph15102091

**Published:** 2018-09-24

**Authors:** Margarita Echeverri, David Anderson, Anna María Nápoles, Jacqueline M. Haas, Marc E. Johnson, Friar Sergio A. Serrano

**Affiliations:** 1 College of Pharmacy, Xavier University of Louisiana; New Orleans, LA 70125, USA; 2 Department of Mathematics, Xavier University of Louisiana; New Orleans, LA 70125, USA; danders2@xula.edu; 3 National Institute on Minority Health and Health Disparities, Bethesda, MD 20892, USA; anna.napoles@nih.gov; 4 Multicultural Community Advisory Board, New Orleans, LA 70118, USA; jacquelineneworleans@gmail.com; 5 African American, Cancer Community Advisory Board, Kenner, LA 70063, USA; mjohnson@fifthcircuit.org; 6 Latino Community Advisory Board, Hispanic Apostolate; Metairie, LA 70003, USA; saserrano@archdiocese-no.org

**Keywords:** cancer research, minorities, cancer health literacy, community-based participatory research, African Americans, Blacks, Hispanics, Latinos, Whites

## Abstract

Although it has been well documented that poor health literacy is associated with limited participation in cancer clinical trials, studies assessing the relationships between cancer health literacy (CHL) and participation in research among diverse populations are lacking. In this study, we examined the relationship between CHL and willingness to participate in cancer research and/or donate bio-specimens (WPRDB) among African Americans, Latinos, and Whites. Participants completed the Cancer Health Literacy Test and the Multidimensional Cancer Literacy Questionnaire. Total-scale and subscale scores, frequencies, means, and distributions were computed. Analyses of variance, the Bonferroni procedure, and the Holm method were used to examine significant differences among groups. Cronbach’s alphas estimated scales’ internal consistency reliability. Significant interactions were found between race/ethnicity, gender, and CHL on WPRDB scales and subscale scores, even after education and age were taken into account. Our study confirms that CHL plays an important role that should be considered and researched further. The majority of participants were more willing to participate in non-invasive research studies (surveys, interviews, and training) or collection of bio-specimens (saliva, check cells, urine, and blood) and in studies led by their own healthcare providers, and local hospitals and universities. However, participants were less willing to participate in more-invasive studies requiring them to take medications, undergo medical procedures or donate skin/tissues. We conclude that addressing low levels of CHL and using community-based participatory approaches to address the lack of knowledge and trust about cancer research among diverse populations may increase not only their willingness to participate in research and donate bio-specimens, but may also have a positive effect on actual participation rates.

## 1. Introduction

The United States is multicultural, and projections indicate that it will become a plurality nation by 2044, meaning that no single racial/ethnic group is projected to be a majority [1]. Currently, Latinos and African Americans are the largest minority groups in the U.S. In 2014, Latinos accounted for 17% (55 M) of the U.S. population and are projected to increase to 29% (119 M) by 2060. African Americans accounted for 13% (42 M) of the U.S. population in 2014 and are projected to increase to 14% (60 M) by 2060 [1]. While African Americans have the highest mortality rate and poorest survival for most major cancers (breast, prostate, colorectal, and lung) [2], Latinos, especially first-generation immigrants, have among the highest incidence and mortality rates for gallbladder, liver, stomach, and cervical cancers [3]. These disparities are partially explained by late-stage diagnosis among African Americans and Latinos [4], and a higher prevalence of infectious agents (HPV, HBV, and the bacterium H. pylori) in Latin American countries [3]. 

Discovery of new methods for cancer prevention, diagnosis, and treatment, and equitable translation of these findings require adequate representation of all major population subgroups in clinical research, including clinical trials. However, low participation rates of minorities in clinical trials contribute to inequities in access to state-of-the-art cancer care and treatments offered in clinical trials [5]. Although many studies have reported low participation of minorities in clinical trials [6,7,8], overall enrollment of African Americans and Latinos in National Institute of Health (NIH) clinical research at U.S. sites has increased over time [9]. The percentage of African Americans enrolled in NIH clinical trials was 9.7% in 2015 and 10.4% in 2016, while they made up 13% of the population. For Latinos, the percentages enrolled were 10.7% in 2015 and 14% in 2016, while they made up 17% of the U.S. population. However, when looking at trials targeting specific health conditions, minority representation is substantially worse. For example, a recent report on clinical trials conducted in the United States during 2015–2016 found that 14.5% of participants were African Americans, but this percentage decreased to 2.7% for cancer-specific trials [10]. A review of 31 studies examining factors influencing the participation of African Americans in cancer clinical trials found that negative beliefs and insufficient knowledge regarding clinical trials are key barriers to participation [11]. Similarly, among Latinos, low levels of knowledge about cancer clinical trials have been reported [12].

Although it has been well documented that poor health literacy is associated with lower utilization of cancer screening examinations and limited participation in cancer clinical trials [5,7,13], there is a lack of health literacy studies that include minority populations [5,7,14]. Misconceptions about scientific research, perceptions of uncertainty and fear of being used as “guinea pigs”, concerns about human subject protections, and cultural factors all contribute to lower rates of participation among African Americans and Latinos [5]. These studies and others highlight the importance of addressing health literacy, scientific literacy, and cultural literacy in educational interventions that aim to promote the participation of minority groups in clinical trials [15]. 

Health literacy has been defined as “the wide range of skills and competencies that people develop to seek out, comprehend, evaluate, and use health information and concepts to make informed choices, reduce health risks, and improve quality of life” [16] (pp.196–197). Based on this definition, Echeverri and colleagues defined cancer health literacy (CHL) as an “individual’s capacity to seek out, comprehend, evaluate, and use basic information and services needed to make appropriate decisions regarding cancer prevention, diagnosis, and treatment” [17] (p. 70). 

Factors that affect individuals’ willingness to participate in cancer research include perceived benefits, barriers, and facilitators as well as sociodemographic characteristics such as age, race/ethnicity, education, and income [18,19,20,21,22]. Studies assessing the relationships between CHL and willingness to participate in research and donate bio-specimens are lacking among minority populations. Consequently, the objective of this study was to examine the relationship between CHL and willingness to participate in cancer research and/or donate bio-specimens for research among African Americans, Latinos, and Whites. Based on the literature, we hypothesized that the level of CHL would be associated positively with the willingness to participate in cancer research and donate bio-specimens.

## 2. Materials and Methods 

### 2.1. Conceptual Framework 

**Figure 1:**
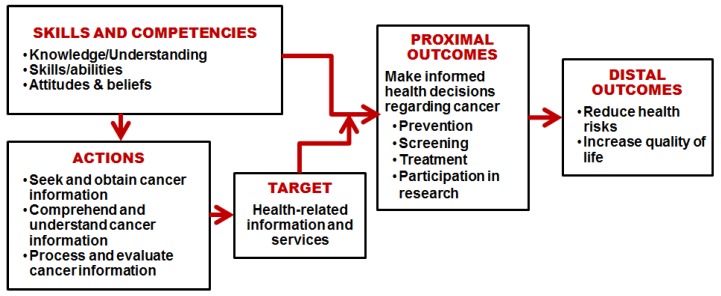
Conceptual framework of the effects of cancer health literacy on cancer-related behaviors and outcomes.

Figure 1 depicts our conceptual framework for this study. CHL implies having the skills and competencies required to seek and understand cancer-related information and risks and make decisions about cancer diagnosis, treatments, and participation in research [17]. As individuals acquire health-related information, they make informed decisions regarding cancer care and participation, ultimately reducing their health risks and improving their quality of life. This multidimensional approach is consistent with other studies recommending the adoption of multilevel approaches to address health literacy barriers and optimize facilitators to enhance minority recruitment for cancer trials [23,24].

### 2.2. Participants and Data Collection Procedures 

This was a community-based participatory research (CBPR) study. Three Community Advisory Boards (CABs), each one representing a different population group in the area (African Americans, Latinos, and Whites), directed community involvement in the project and made decisions about project aims, budget allocation, and dissemination of the results. Each CAB worked with a community outreach facilitator and community assistants who distributed flyers and scheduled events to screen; registered and obtained consent from study participants; administered surveys at community sites; and entered survey data into the system. The study was approved by Xavier University of Louisiana’s Internal Review Board, Study Numbers #511 (Latinos), #540 (African Americans), and #587 (Whites) (Institutional Review Board (IRB): Assurance of Compliance No. FWA 00004443). Oral informed consent was obtained and completing the questionnaires indicated consent to take part in the study. A script of the oral consent information was also given to each participant before accepting to participate. The sample was stratified by race/ethnicity (Latino, Non-Latino African American, and Non-Latino White) and gender (male/female). 

### 2.3. Instruments 

Participants completed a questionnaire that included the Cancer Health Literacy Test (CHLT-30) [25]. Spanish (CHLT-30-DKspa) and English (CHLT-30-DKeng) versions of the CHLT-30 (Cronbach Alpha = 0.88) were used. Because the Spanish version included a “don’t know” (DK) response option for each question [17], we added the same DK response option to the English version and pilot-tested it among 24 English-speaking members of the African American and White communities (12 in each community). The CHLT-30 includes 30 multiple-choice questions related to different domains of cancer health literacy (knowledge, reading, and numeracy), where only one response option is correct for each item. CHL scores were calculated as the count of correct answers to the CHLT-30 (range 0 to 30). DK answers were coded as wrong, thus higher scores = higher CHL.

Additionally, participants completed the Multidimensional Cancer Literacy Questionnaire (MCLQ), a new tool that was developed for this study. The MCLQ is divided into several components related to cancer health literacy (cancer history, cancer screening, barriers and facilitators to screening, cultural beliefs and perceptions about cancer, perceived cancer risks, trust in healthcare providers, and perceptions of cancer research). This study focuses only on the MCLQ scale that assesses perceptions of cancer research named the Willingness to Participate in Research and Donate Bio-specimens (WPRDB) Scale (Cronbach Alpha = 0.95). These questions were taken as is or adapted, with author permission, from a scale developed by the National Cancer Institute Geographic Management of Cancer Health Disparities Program (GMaP)-Region 2 (formerly Region 3), and lead by Dr. Isabel Scarinci in 2013 (unpublished). 

The WPRDB included 21 questions organized into three domains, see Table 1. The first domain, Willingness to Participate in Research Studies (*WILL:Research*), contained six questions on willingness to participate in research studies that involve completing: A survey or interview about cancer knowledge, attitudes, and practices; participating in cancer education sessions; take an experimental drug that may prevent/cure cancer; take natural supplements; undergo a minor procedure or test; or undergo a major procedure that may require hospitalization. The second domain, Willingness to Donate Bio-specimens for Research (*WILL:Donate*), included six questions focused on the willingness to: Donate saliva; donate cheek cells; provide a urine sample; donate a blood sample; donate a skin sample; and donate a tissue sample. The third domain, Willingness to Participate in Cancer Studies by Institutions Conducting the Research (*WILL:Institution*), contained nine questions that focused on willingness to participate in research studies that are conducted by: Their personal physicians; a university medical school or hospital; a local hospital or clinic that is not affiliated with a university; a governmental agency at the federal, state, or city level; a non-profit organization (such as a foundation or charity organization); a for-profit business; a tobacco company; a pharmaceutical company; or an insurance company. A four-level response set was used for all items: 1 = very unlikely; 2 = somewhat unlikely; 3 = somewhat likely; or 4 = very likely.

Demographic variables included age (categorized as 25–40; 41–54; or 55+), gender (female or male) and educational level (categorized as 8th grade education or less; some high school; high school diploma; some college or vocational studies; or a bachelor’s or more advanced degree). 

### 2.4. Statistical Analysis

Descriptive statistics reported include frequencies, means, and distributions. The WPRDB total-scale score and the subscale scores were calculated as the mean of all non-missing answers. Analyses of variance (ANOVAs) were conducted to test for significant differences in CHL scale scores and responses to the individual WPRDB items and scale scores by race/ethnicity, gender, age, and educational level. The Bonferroni procedure and the Holm method were utilized to examine post hoc analysis of the differences between the groups. Cronbach's alphas were used to assess the reliability of the WPRDB total scale and its three subscales (domains). Analyses of variance were used to analyze the relationships between CHL-scores and predicted values of WPRDB scale and subscale mean scores, controlling for demographic variables. R and SPSS, version 19 (SPSS Inc., Chicago, IL, USA) were used to carry out the data analyses.

## 3. Results

A total of 1500 participants (500 Latinos, 500 non-Latino African Americans, and 500 Non-Latino Whites) completed the questionnaires (CHLT and MCLQ). All Latinos completed the surveys in Spanish while African Americans and Whites completed them in English. Half of participants were women and the mean age was 48.3 years (SD = 14.9; range = 25 to 94). In general, participants were roughly equally distributed by age, although Latinos were somewhat younger and less educated than their counterparts, see Table 2. 

### 3.1. Cancer Health Literacy (CHL) Scores 

**Figure 2:**
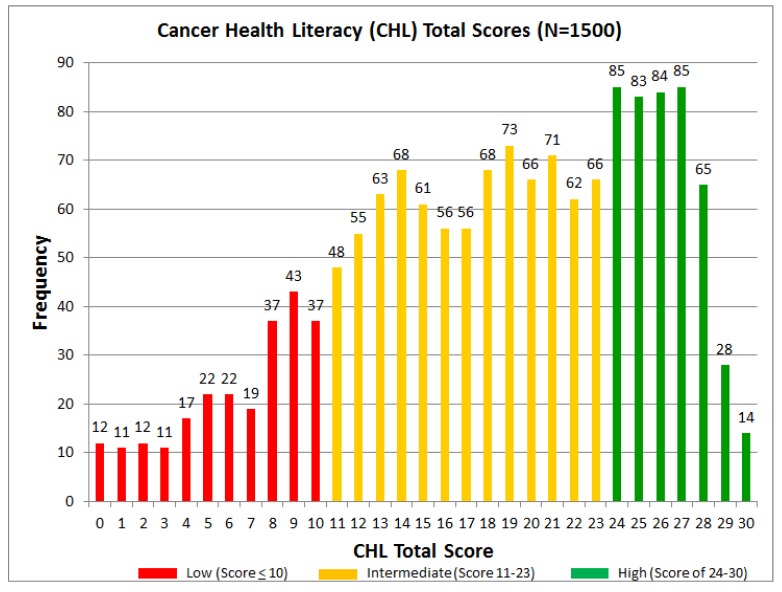
Cancer health literacy total scores.

**Figure 3:**
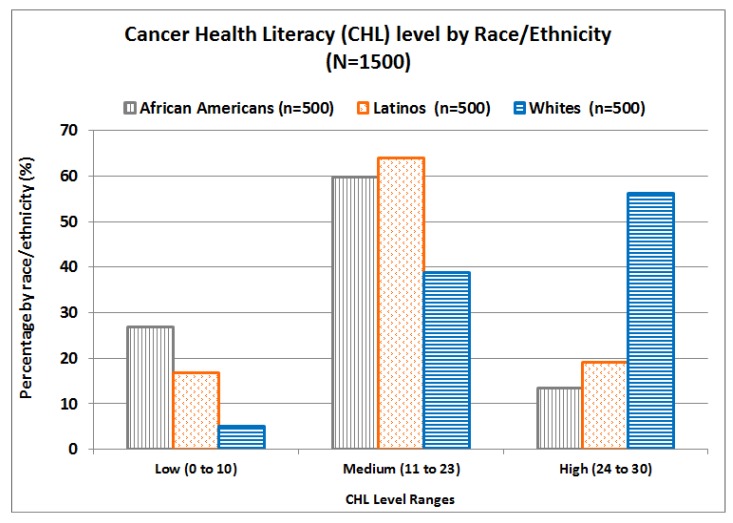
Cancer health literacy levels by race/ethnicity.

The mean CHL-score for the entire sample (N = 1500) was 18.16 (range 0 to 30, SD = 7.19). While 14 people had the maximum score of 30 points, 12 participants had the minimum score of 0 points; thus, the entire range was observed. Based on CHL-total scores, shown in Figure 2, three main groups were identified: 16.2% of participants were classified in the low CHL level (score of 10 or lower), 54.2% in the medium level (scores 11 to 23), and 29.6% in the high level (scores 24 to 30). Figure 3 shows the percentages of African Americans, Latinos, and Whites in each CHL-level. In general, Whites had higher CHL-levels, while Latinos had higher levels than African Americans.

**Figure 4:**
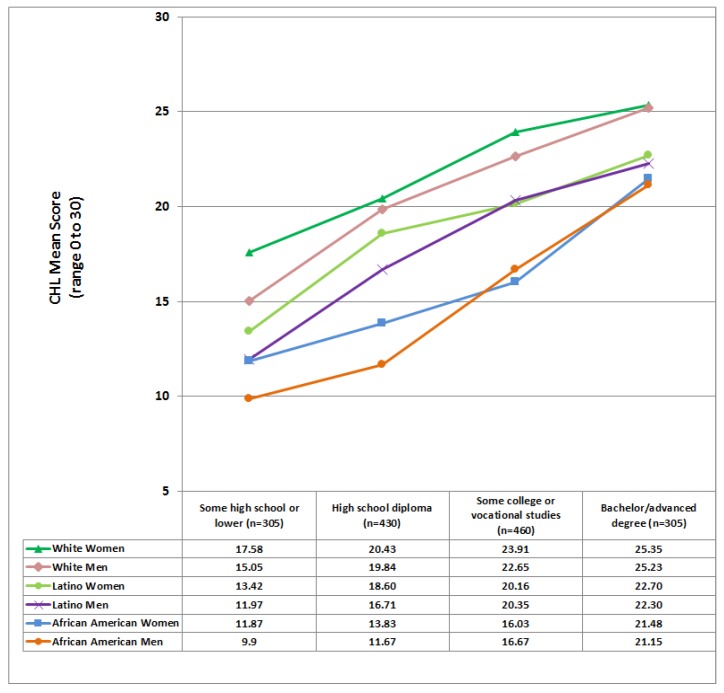
Cancer Health Literacy (CHL) mean scores by race/ethnicity, gender, and education. Note: the group with an education lower than primary school (*n* = 126) and the group with some high school education (*n* = 179) in Table 1 were combined because of their small sample size when compared to the other three educational groups.

Significant differences were found in CHL-scores by race (*p* < 0.0001), gender (*p* < 0.0008), and education (*p* < 0.0001), but not by age. In general, Whites had a significantly higher mean CHL-score (M = 22.52) than the other groups (*p* < 0.0001). However, African Americans had a significantly lower mean CHL-score (M = 14.78) than Latinos (M = 17.17), and Latinos had a significantly lower mean CHL-score than Whites. Interestingly, mean CHL-scores for women (M = 18.72) were significantly higher (*p* = 0.0008) than for their male counterparts (M = 17.6). As expected, each increase in educational level was associated with a statistically significant increase in CHL-scores (*p* < 0.0001): The mean CHL-scores for each education level were: Primary school or less, M = 10.83; some high school, M = 13.33; high school diploma; M=16.31; some college or vocational studies, M = 20.11; and a bachelor or advanced degree, M = 23.69. A statistically significant race/ethnicity and education level interaction was found: Although CHL-mean scores increased with educational level, this increase differed by demographic group, see Figure 4. In general, as seen in Figure 4, women tended to score higher than men for every racial/ethnic group.

### 3.2. Willingness to Participate in Research and Donate Bio-Specimens (WPRDB) Scores

Overall, the mean total WPRDB scale score was 2.46 (range 1 to 4), see Table 1. However, the willingness to donate bio-specimens (*Will-Donate*) subscale had the highest mean score of the three subscales, see Table 1, meaning that participants were more willing to donate bio-specimens (M = 2.76) than participate in research studies (M = 2.44). In general, those self-reporting as Latinos had higher means on all (total and subscale) scores than their counterparts, see Table S1. Interestingly, no differences were found by gender on any scale/subscale scores. By age, the only significant difference found was that participants older than 55 years had higher *Will-Donate* mean subscale scores than those under 40, see Table S1.

**Figure 5:**
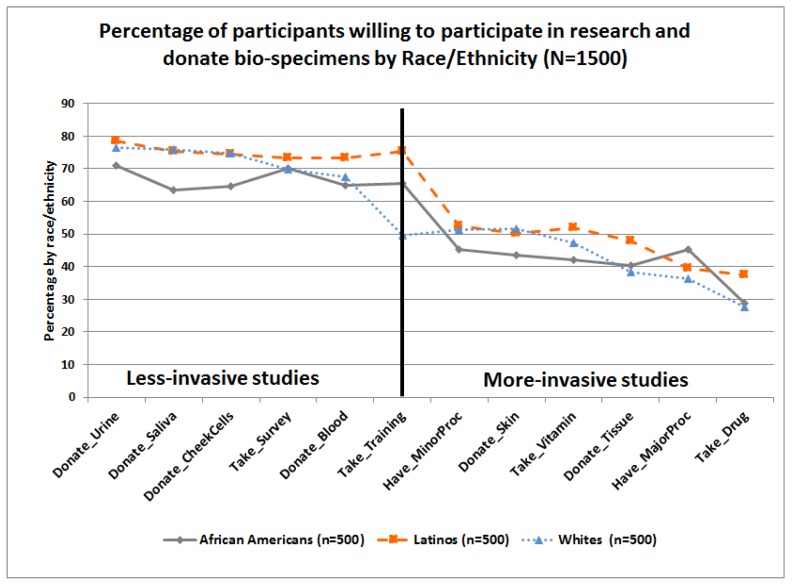
Item-level differences on willingness to participate in research and donate bio-specimens scale by race/ethnicity.

**Figure 6:**
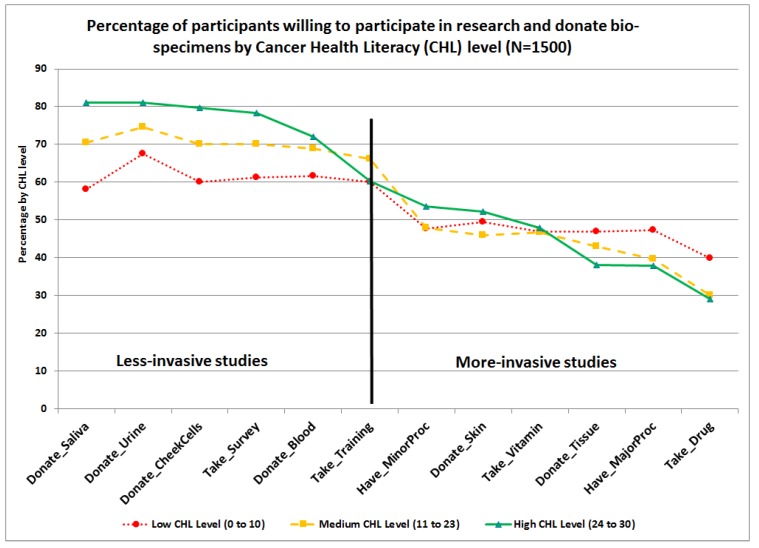
Item-level differences on willingness to participate in research and donate bio-specimens scale by cancer health literacy level.

Percentages of participants who answered the WPRDB questions in a positive way, that is, “somewhat likely” or “very likely” to participate in research studies (*WILL:Research*) and/or donate bio-specimens for research (*WILL:Donate*) are presented in Figure 5, by race/ethnicity. As observed, the three groups have similar trends. Higher percentages of respondents were willing to participate in less-invasive studies requiring completing a survey, attending training, and/or donating urine, saliva, cheek cells, and blood. However, lower percentages were willing to participate in more invasive research studies requiring donating skin or tissue, taking an experimental drug or nutritional supplement such as vitamins with unknown benefits and secondary effects, and/or undergoing a minor or major procedure that requires exposure to radiation or hospitalization. Percentages of participants who answered the WPRDB questions in a positive way were also presented by CHL-levels, see Figure 6. Those with high CHL-level had higher means associated with their willingness to participate in research requiring them to complete a survey (M = 3.10), or donate samples of saliva (M = 3.14), check cells (M = 3.12), urine (M = 3.14), and skin (M = 2.53), see Table S1. 

### 3.3. Willingness to Participate by Institution Type (WILL:Institution)

**Figure 7:**
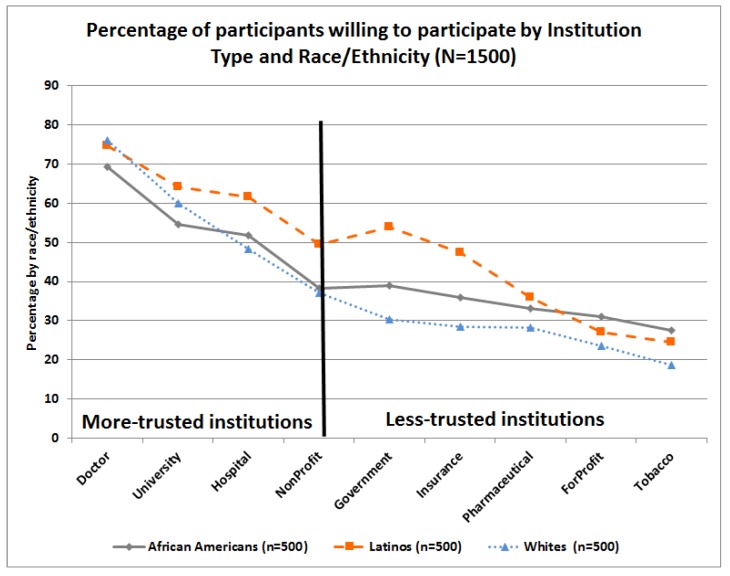
Item-level differences on willingness to participate in research by institution type scale and race/ethnicity.

**Figure 8:**
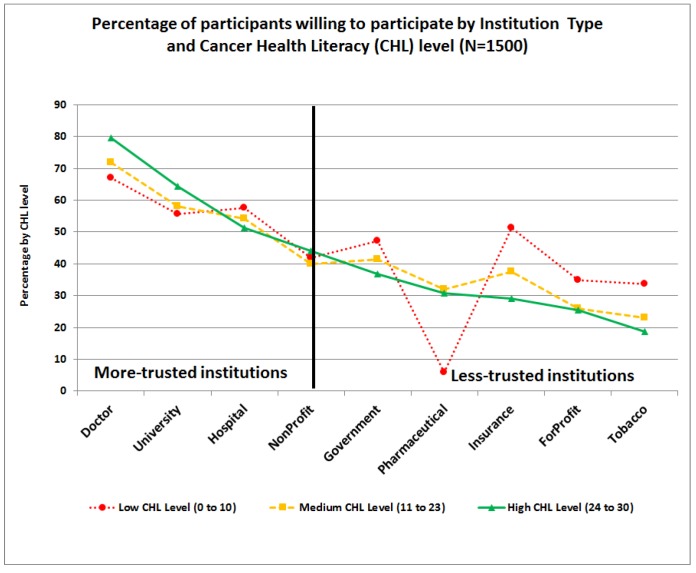
Item-level differences on willingness to participate in research by Institution Type scale and Cancer Health Literacy level.

This subscale had the lowest mean score (M = 2.26) of the three subscales, see Table 1. At the item level, higher mean scores were observed for participating in research conducted by their own doctor and lower scores for participating in studies conducted by for-profit and tobacco companies, see Table 1. Significant differences were found by age and race/ethnicity. In general, Latinos had significantly higher mean subscale scores than their counterparts and participants older than 55 years had significantly lower mean subscale scores than those under 40 years. Similarly, questions in the willingness to participate by institution type (*WILL:Institution*) scale were classified by race/ethnicity, see Figure 7, and by CHL-Level, see Figure 8. As observed, higher percentages of participants were willing to participate in research studies conducted by institutions that are more familiar or trusted, such as research conducted by their own doctors or local universities or hospitals, and lower percentages of participants were willing to participate in research conducted by organizations that have more controversial public images, e.g., tobacco companies.

In general, Latinos were more likely to indicate a willingness to participate in almost all items of the institution type (*WILL:Institution*) subscale, compared to their counterparts, see Figure 7. Those with low CHL-level were significantly more willing to participate than their counterparts with higher CHL in research conducted by insurance companies (M = 2.42), for-profit organizations (M = 2.03), and tobacco manufacturers (M = 1.95), but not by pharmaceutical companies (M = 2.09), see Figure 8. In contrast, those with high CHL were significantly more likely to indicate a willingness to participate in research conducted by their own doctors (M = 3.05) or a university (M = 2.77), see Table S1. 

### 3.4. Interactions Between Cancer Health Literacy, Race/Ethnicity, and Gender on Total WPRDB Scale 

**Figure 9:**
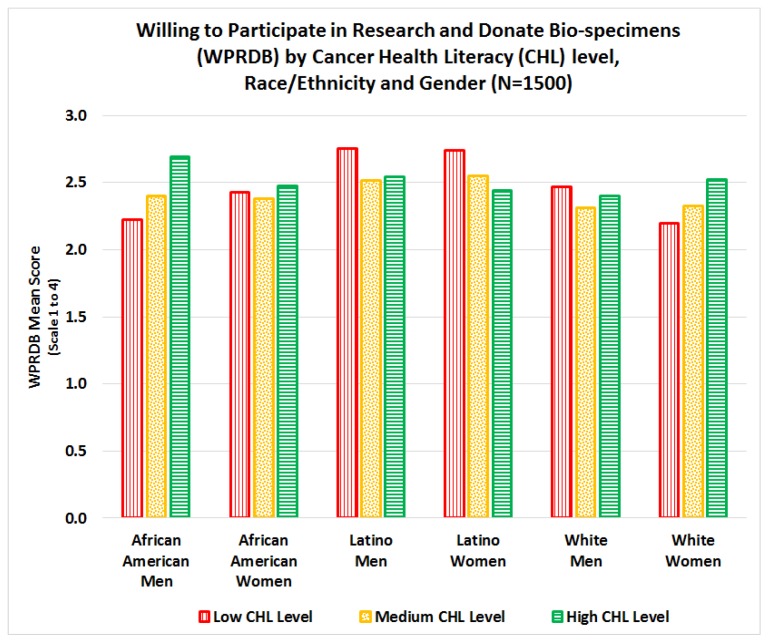
Interaction between cancer health literacy, race/ethnicity, and gender on WPRDB total scale.

While WPRDB mean scale scores varied by demographic characteristics, the effect sizes were fairly small. Less than 10% of the variability in WPRDB mean scores was explained by demographic variables and CHL scores. In contrast, almost half (44%) of the variability in CHL scores was explained by demographic variables. In general, Latinos had significantly higher total WPRDB scores than their counterparts (*p* = 0.0027). A significant interaction (*p* = 0.0135) was found between CHL score, race/ethnicity, and gender on total WPRDB mean scale score, see Figure 9. For African American men and White women, mean WPRDB score increases as CHL score increases; while for Latino men and women, mean WPRDB score decreases as CHL score increases. 

**Figure 10:**
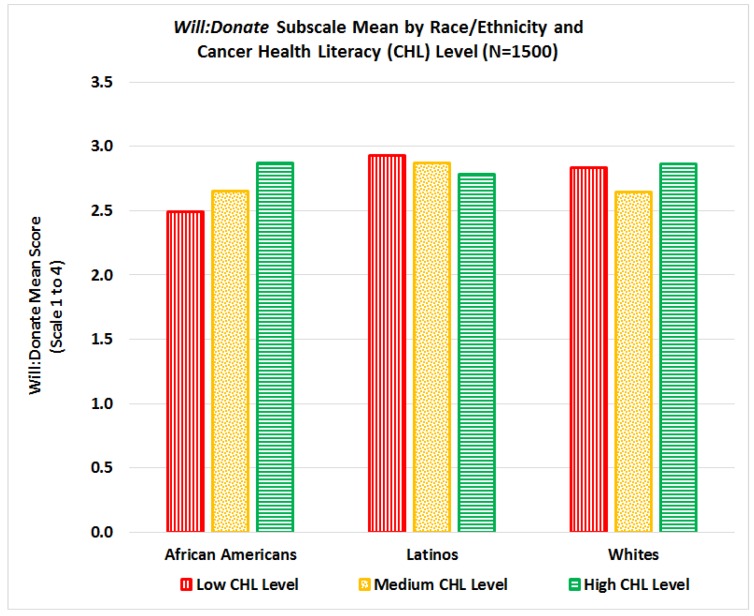
Interaction between cancer health literacy and race/ethnicity on *Will:Donate* subscale mean score.

When conducting separate analyses on the interactions between the different subscales, we found that the CHL-score has no significant impact on the willingness to participate in research studies subscale (*WILL:Research*), even when interactions by race/ethnicity, education or age are considered. For the willingness to donate bio-specimens for research (*WILL:Donate*), African Americans had significantly lower mean scores than Latinos (*p* = 0.0034) and Whites (*p* = 0.078), but no significant differences were found between Latinos and Whites. However, we found a significant interaction between race/ethnicity and CHL scores on *Will:Donate* mean scores (*p* = 0.0015) indicating that *Will:Donate* mean scores for African Americans increase as CHL scores increase, for Latinos mean scores decrease as CHL increases, and for Whites *Will:Donate* mean scores decrease only for those in the medium level of CHL, see Figure 10.

### 3.5. Interactions in willingness to participate by institution type (WILL:Institution)

**Figure 11:**
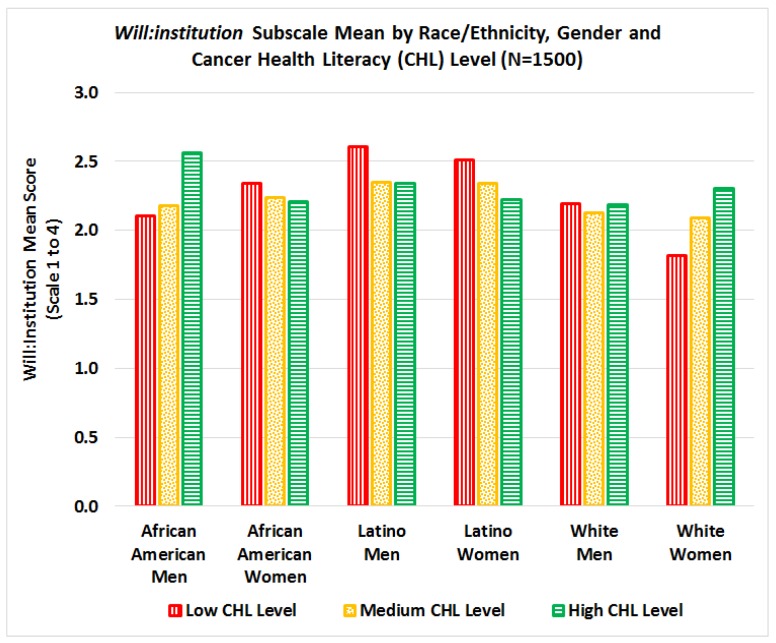
Interaction between cancer health literacy, race/ethnicity, and gender on *Will:Institution* subscale mean scores.

In general, Latinos had a significantly higher *Will:Institution* mean score than their counterparts (*p* = 0.03 for African Americans and *p* = 0.0013 for Whites). A significant interaction (*p* = 0.0154) was found between CHL score, race/ethnicity, and gender on *Will:Institution* mean scale score, see Figure 11. For African American men and White women, mean *Will:Institution* scores increase as CHL scores increase, while for Latino men/women and African American women, mean *Will:Institution* scores decrease as CHL scores increase.

## 4. Discussion

This study examined relationships between cancer health literacy levels and willingness to participate in research and donate bio-specimens (WPRDB) for cancer research, stratified by race/ethnicity and gender. Although most literature focuses on racial/ethnic differences in willingness to participate in cancer research and donate bio-specimens, our results indicate that cancer health literacy (CHL) is an important factor that should be included in the equation. Specifically, in this study we found three significant interactions: 1) significant interactions between CHL, race, and gender for total WPRDB scale score, see Figure 9; 2) significant interactions between CHL and race for willingness to donate bio-specimens for research (*Will:Donate*), see Figure 10; and 3) significant interactions between CHL, race, and gender on willingness to participate by type of institution conducting the study (*Will-Institution*), see Figure 11. Although we hypothesized that the level of CHL would be *positively* associated with willingness to participate in cancer research and donate bio-specimens, this relation was not always positive, see Figure 9. While there was no interaction between CHL and willingness to participate in research (*Will:Research*), the relationship between CHL scores and willingness to donate bio-specimens (*Will:Donate*) was positive for African Americans, negative for Latinos, and not clear for Whites, see Figure 10. The relationship between CHL score and willingness to participate in studies by type of institution was positive for African American men and White women, but negative for African American women and Latino men/women, as shown in Figure 11. Thus, conducting more research on the relationship among CHL and willingness to participate in cancer research and bio-banking studies among all groups is advisable.

In summary, in the total sample, willingness to participate was over 60% for less invasive research studies (i.e., complete a survey, give a sample of saliva, provide cheek cells, and provide a urine sample). Over half of participants were willing to participate if the study is conducted by their own doctor, a university, or a hospital, and less willing to participate if the study is conducted by a government agency; non-for-profit organization; health insurance organization; pharmaceutical company; for-profit organization; or a tobacco industry entity. Latinos were more likely to be willing to participate in cancer research and/or donate bio-specimens than African Americans or Whites, after controlling for gender, age, and educational level (*p* < 0.01). 

Our findings that minorities are willing to participate in cancer research are consistent with a study that demonstrated, in a review of over 70,000 individuals and 20 studies, that consent rates among White, African American, and Latino participants were similar. That study concluded that “racial and ethnic minorities in the US are as willing as non-Latino whites to participate in health research” [26] (p.201). However, Byrne [27] stated that there are important differences between “willing to participate” and “real participation” and found that even though African American, Latino, and White cancer patients were equally likely to participate in cancer trials, non-English speaking Latinos were less likely to have informed participation because of language barriers. Language barriers can be addressed by making research tools available in different languages and using trained interpreters or bilingual research personnel [28,29]. In our case, Latinos completed the tools in Spanish and researchers and CAB members were fluent in both English and Spanish. These strategies may have had a positive impact on our results with Latino participants reporting significantly higher scores on willingness to participate and donate bio-specimens for research than their counterparts. Unfortunately, English fluency as an eligibility criterion to participate in clinical trials has increased in recent years [30,31], which may be against the Principle of Justice established in the Belmont Report [32] and Common Rule [33] requiring fair and appropriate participation of vulnerable populations. Excluding participants simply on the basis of language is questionable for ethical reasons and also may introduce bias in research studies that are reporting low willingness to participate among minority populations without controlling for language differences. The genetic and health profiles of immigrant and non-immigrant populations differ on many health indicators; thus, it is important that non-English-speaking groups have access to and participate in cancer clinical trials.

Another important barrier identified across race/ethnicity is the lack of trust in biomedical research [18,22,34,35,36]. Kerasidou [34] clearly argued that although informed consent and oversight by research ethics committees and institutional review boards play an important role in increasing public trust, professional integrity is the key to promoting participants’ trust and real participation. Our study confirms that the type of institution conducting the research may play a crucial role in willingness to participate, and these effects vary by race/ethnicity and gender, see Figure 11. We found that participants were more willing to participate in research conducted by their own doctors, and hospitals and universities, but remain skeptical towards research conducted by pharmaceutical, for-profit, tobacco, and insurance companies, see Figure 7 and Figure 8. Although all institutions are required to protect participant privacy, autonomy, and safety and comply with the same ethical regulations throughout the research process (recruitment, interventions, data collection, publications of results, etc.), some studies have found that research participants may place more trust in those who they know are responsive to their vulnerability and have a “good will” towards them [34,37]. Thus, physician attitudes toward cancer research and the impact of new treatments on their patients are key in clinical trial participation [18].

Other barriers to increasing the participation of minorities in cancer research are focused on cultural barriers and participants’ lack of knowledge regarding research and clinical trials [22,27,38,39,40,41]. While a commonly reported barrier for bio-specimen donation among African Americans was poor knowledge about bio-banking and lack of clarity about how bio-specimens will be used in research [42], others have focused on the lack of access to health research among all groups [18,26] or on the effects of restrictive eligibility criteria among a single racial/ethnic group [43]. Regardless of the different barriers, there is a general consensus in the literature that educational interventions among diverse communities are key to address these barriers [44,45,46,47], and that community involvement has a positive impact in participant enrollment in research studies [45,48,49,50]. As a result of using a community-based participatory research (CBPR) approach, in our study we were able to exceed our targeted recruitment goals by 67% for Latinos (from 300 to 500) and 25% for African Americans (from 400 to 500). Among the main CBPR strategies used in our study was the pre-existence of trustful relationships with the members of the Community Advisory Boards (CABs) in each target population (African Americans, Latinos, and Whites). These members not only were leaders in their respective communities but also were very interested in conducting the research. As part of the project, they received training regarding health disparities in their communities, learned more about research processes and bio-banks; participated in the development of the research proposal including recruitment strategies; made decisions about hiring community assistants and budget allocations; and conducted community cancer forums to disseminate study results. Thus, using a community-based approach to increase awareness and knowledge regarding cancer risks and willingness and real participation in cancer research and bio-banking studies is highly recommended and could help explain the willingness of certain traditionally overlooked groups, e.g., Latinos, to participate. 

The finding that the willingness to donate bio-specimens among African Americans increased as CHL increased, while for Latinos willingness to donate bio-specimens decreased as CHL increased, see Figure 10, is intriguing. Perhaps this signifies that among African Americans, a group who traditionally has had greater exposure to research with some of these experiences being highly publicized exploitive studies, e.g., Tuskegee, increasing knowledge about cancer is a way to foster trust and interest in donating specimens. Among Latinos, a group that has traditionally been overlooked by research and excluded based on the lack of language assistance provided by trials, once they learn more about cancer, this may raise more questions that need to be addressed prior to their agreeing to donate samples. Both of these interpretations suggest that sensitivity to historical experiences of groups with research is critical, and that targeted approaches to enhance bio-specimen collection may be necessary.

## 5. Conclusions

In summary, significant differences in the willingness to participate or donate bio-specimens for research were found by CHL level and race/ethnicity and gender, even after education and age were taken into account. Our study confirms that CHL plays an important role that should be considered in the discussion of minorities’ participation in cancer research and more specifically in clinical trials. The majority of participants, regardless of race/ethnicity, were more willing to participate in *non-invasive* research studies (surveys, interviews, and training) or collection of bio-specimens (saliva, check cells, urine, and blood) and in studies led by their own healthcare providers, and local hospitals and universities. However, participants were less willing to participate in *more*-*invasive* studies requiring them to take medications, undergo medical procedures, or donate skin/tissues. The type of institution conducting the research was also associated with the participants’ willingness to participate in studies and donate bio-specimens. 

Our results are the first, to our knowledge, advancing research on measures of cancer health literacy and willingness to participate in cancer research for these three population groups. Results support the use of the CHLT-30 and the WPRDB questionnaire (English and Spanish versions) among African Americans, Latinos (English- and Spanish-speaking), and Whites to measure cancer literacy and examine cancer-related attitudes and behaviors regarding cancer research. Considering the interactions found between CHL, race/ethnicity, and gender, we recommend that our findings and tools be reviewed and applied in other studies to advance the field. Measuring CHL and WPRDB should be the first step when developing informational resources on cancer research and bio-banking aimed to improve rates of participation among diverse racial/ethnic and gender populations. Combining such informational programs and CBPR approaches to address the lack of knowledge and trust regarding cancer research among all populations may increase not only their willingness to participate in studies and donate bio-specimens, but may also contribute to the increase of actual participation rates.

## Figures and Tables

**Table 1:** Willingness to Participate in Cancer Research and Donate Bio-specimens (WPRDB) Scale ^1^ among African Americans, Latinos, and Whites in Louisiana (N = 1500).

Domain/Item Description	Number of Items	Mean ^2^	Std. Deviation	Variance	Cronbach’s Alpha
**Domain 1: Willingness to participate in research studies (*WILL:Research*)**	**6**	**2.44**	**0.831**	**0.690**	**0.867**
Complete a survey or interview that asks about your behaviors, beliefs and practices (for example, eating habits, physical activity, getting your regular checkups and recommended cancer screenings)		2.92	1.019	1.038	
Attend a number of educational sessions or classes such as a program about healthy eating or cancer screenings		2.76	1.017	1.034	
Take an experimental drug (e.g., pills), for example, a drug to test whether it could prevent a particular type of cancer		2.02	1.043	1.087	
Take experimental natural supplements (e.g. vitamins)		2.34	1.090	1.187	
Undergo a minor experimental procedure or test (such as an X-ray) that does not require hospitalization, does not require you be put to sleep, or does not cause you any pain		2.38	1.130	1.277	
Undergo a major procedure (such as a test to detect colon cancer early) that may involve hospitalization or being put to sleep		2.21	1.130	1.276	
**Domain 2: Willingness to donate bio-specimens for research (*WILL:Donate*)**	**6**	**2.76**	**0.957**	**0.916**	**0.932**
Give a sample of your saliva or spit		2.97	1.074	1.154	
Allow researchers to rub the inside of your mouth with a Q-tip to collect a sample of your cheek cells		2.95	1.071	1.148	
Provide a sample of your urine		3.05	1.037	1.075	
Allow researchers to collect about 1 tablespoon of your blood		2.89	1.107	1.225	
Allow researchers to collect a tiny piece of your skin, about the size of a pencil eraser and using numbing medicine to prevent you from feeling pain		2.42	1.179	1.390	
Allow researchers to collect a small piece of tissue the size of a pencil eraser (called a biopsy) from one of your organs, such as your liver or breast, and using numbing medicine to prevent pain		2.28	1.173	1.376	
**Domain 3: Willingness to participate in a cancer study by type of institution conducting the research (*WILL:Institution*)**	**9**	**2.26**	**0.825**	**0.681**	**0.927**
Study conducted by your own doctor		2.95	1.018	1.037	
Research study conducted by a university medical school or university hospital		2.63	1.050	1.103	
Research study conducted by a local hospital or clinic that is not affiliated with a university		2.52	1.062	1.129	
Research conducted by a governmental agency at the federal, state or city level		2.24	1.060	1.124	
Research conducted by a non-profit organization (such as a foundation or charity organization)		2.23	1.050	1.103	
Research conducted by a for-profit business (such as private organizations providing healthcare services to make money)		1.91	0.989	0.978	
Research conducted by a company that produces tobacco-related products (e.g., cigarettes, cigars, and e-cigarettes)		1.79	0.988	0.976	
Research conducted by a pharmaceutical company (such the ones that produce medications)		2.00	1.036	1.074	
Research conducted by a health insurance company		2.11	1.083	1.173	
**WPRDB Total Scale Score**	**21**	**2.46**	**0.746**	**0.557**	**.948**

^1^ Questions copied/adapted, with permission, from a scale developed by the National Cancer Institute Geographic Management of Cancer Health Disparities Program (GMaP)-Region 2 (formerly Region 3), and lead by Dr. Isabel Scarinci in 2013 (unpublished). ^2^ Response options were 1 = very unlikely; 2 = somewhat unlikely; 3 = somewhat likely; 4 = very likely.

**Table 2:** Demographic characteristics of participants.

	Non-Latino African Americans		Latinos (Any Race)		Non-Latino Whites		Total	
	*n*	%	*n*	%	*n*	%	*n*	%
**Total Participants**	500	33.3	500	33.3	500	33.33	1500	100.0
**Gender**								
Male	250	50.0	250	50.0	250	50.0	750	50.0
Female	250	50.0	250	50.0	250	50.0	750	50.0
**Age**								
25–40	135	27.0	211	42.2	181	36.2	527	35.1
41–55	169	33.8	150	30.0	143	28.6	462	30.8
56+	196	39.2	139	27.8	176	35.2	511	34.1
**Education**								
Primary school or less	11	2.2	112	22.4	3	0.6	126	8.4
Some High School	76	15.2	74	14.8	29	5.8	179	11.9
High School Diploma	187	37.4	111	22.2	132	26.4	430	28.7
Some college studies	152	30.4	133	26.6	175	35.0	460	30.7
Bachelor or advanced degree	74	14.8	70	14.0	161	32.2	305	20.3

## Supplementary Materials

The following are available online at http://www.mdpi.com/1660-4601/15/10/2091/s1, Table S1: Mean differences in Cancer Health Literacy (CHT), and Willing to Participate in Research and Donate Bio-specimens (WPRDB) scores by demographic characteristics.
