# Supplementary material for: Cancer Health Literacy and Willingness to Participate in Cancer Research and Donate Bio-Specimens
Source: Int J Environ Res Public Health. 2018 Sep 24;15(10):2091. doi: 10.3390/ijerph15102091 (PMC6211072; doi:10.3390/ijerph15102091)
Supplement: Supplementary file 1 [file ijerph-15-02091-s001.pdf]

Table S1. Mean differences in Cancer Health Literacy (CHL)* and Willing to Participate in Research and Donate Bio-specimens (WPRDB) scores by demographic characteristics.**

Domains and Items	Education						Gender			Race/Ethnicity				Age				CHL Level			
	p	Primary school or lower	Some High School	High School Diploma	Some college studies	Bachelor/ advanced degree	*p*	**Men**	**Women**	*p*	**Blacks**	**Hispanics**	**Whites**	*p*	**25-40**	**41-55**	**55+**	*p*	**Low (0 to 10)**	**Medium (11 to 23)**	**High (24 to 30)**
**Total participants**		126	179	430	460	305		750	750		500	500	500		527	462	511		243	813	444
**CHL-Score (*)**	<.000	10.83 c	13.33 c	16.31 c	20.11 c	23.69 c	.008	17.60	18.72a	<.000	14.78c	17.17c	22.52c	.520	18.13	18.45	17.92	<.000	6.44c	17.25c	26.23c
WILL:Research	<.000	2.66a	2.51	2.31b	2.43	2.49	.521	2.42	2.45	0.001	2.40	2.55a	2.36	.094	2.38	2.44	2.49	.306	2.50	2.41	2.45
R3a_Survey	<.000	2.98	2.98	2.70b	2.93	3.17	.072	2.88	2.97a	0.064	2.88	3.01	2.88	.717	2.90	2.92	2.95	<.000	2.77	2.87	3.10a
R3b_Training	<.000	3.16a	2.88	2.60b	2.76	2.77	.016	2.70	2.83a	<.000	2.77c	3.06c	2.46c	.185	2.70	2.76	2.82	.194	2.72	2.81	2.71
R3c_Drug	<.000	2.33a	2.20	1.96	1.97	1.94	.276	2.05	1.99	0.002	1.95	2.15a	1.95	.878	2.01	2.01	2.04	.001	2.25a	1.98	1.97
R3d_Vitamin	.002	2.57a	2.43	2.18b	2.36	2.38	.356	2.31	2.36	0.005	2.22	2.45a	2.34	.607	2.30	2.36	2.36	.247	2.42	2.30	2.36
R3e_MinorProc	.082	2.57a	2.35	2.27b	2.40	2.42	.837	2.38	2.37	0.091	2.29	2.44	2.40	.134	2.30	2.39	2.44	.247	2.42	2.33	2.44
R3f_MajorProc	.525	2.32	2.21	2.15	2.18	2.27	.522	2.22	2.19	0.033	2.31a	2.16	2.14	.000	2.07b	2.20	2.35a	.006	2.42a	2.18	2.14
WILL:Donate	<.000	2.91	2.69	2.58b	2.80	2.92	.550	2.74	2.77	0.001	2.64b	2.86	2.78	.005	2.67	2.74	2.86a	.046	2.67	2.74	2.85a
R4a_Saliva	<.000	3.08	2.87	2.74b	3.01	3.23a	.532	2.95	2.98	<.000	2.77b	3.09	3.04	.017	2.90	2.92	3.07a	<.000	2.74c	2.94c	3.14a
R4b_Cheek	<.000	3.09	2.80	2.74b	3.01	3.20a	.563	2.93	2.97	<.000	2.80b	3.04	3.02	.016	2.89	2.90	3.06a	<.000	2.76	2.92	3.12c
R4c_Urine	<.000	3.25a	2.97	2.88b	3.08	3.23	.384	3.03	3.07	0.002	2.94	3.16a	3.05	.009	2.98	3.00	3.16a	.023	2.92	3.04	3.14a
R4d_Blood	.001	3.09a	2.87	2.72b	2.92	3.02	.192	2.85	2.93	0.003	2.79	3.03a	2.86	.005	2.80	2.86	3.02a	.247	2.80	2.89	2.95
R4e_Skin	.003	2.50	2.34	2.26b	2.46	2.59a	.369	2.45	2.39	0.015	2.29b	2.47	2.49	.009	2.30	2.43	2.53a	.037	2.42	2.36	2.53a
R4f_Tissue	.057	2.47a	2.27	2.16b	2.34	2.27	.468	2.25	2.30	0.021	2.24	2.39a	2.19	.038	2.17b	2.32	2.35	.092	2.41a	2.27	2.21
WILL:Institution	<.000	2.51a	2.20	2.13b	2.29	2.34	.816	2.26	2.27	0.002	2.24	2.37a	2.19	.022	2.32	2.28	2.19b	.341	2.32	2.24	2.28
R5a_Doctor	<.000	3.16a	2.77b	2.82	2.98	3.12	.020	2.89	3.01a	0.099	2.87	3.01	2.98	.053	2.87	3.02	2.98	.033	2.87	2.92	3.05a
R5b_University	<.000	2.79	2.53	2.43b	2.68	2.84a	.476	2.61	2.65	0.002	2.53	2.76a	2.61	.852	2.63	2.66	2.62	.004	2.53	2.59	2.77a
R5c_Hospital	<.000	2.86a	2.36	2.37	2.56	2.65	.662	2.54	2.51	<.000	2.45	2.71a	2.41	.303	2.55	2.56	2.47	.809	2.53	2.51	2.55
R5d_Government	<.000	2.60a	2.25	2.07	2.26	2.28	.330	2.26	2.21	<.000	2.17	2.49a	2.05	.010	2.32	2.26	2.13b	.381	2.32	2.23	2.20
R5e_NonProfit	<.000	2.53a	2.14	2.02b	2.27	2.39	.507	2.25	2.21	<.000	2.13	2.38a	2.18	.116	2.29	2.25	2.15	.123	2.19	2.20	2.32
R5f_ForProfit	.129	1.99	1.83	1.83	1.94	1.99	.958	1.91	1.91	0.055	2.00	1.85	1.88	.006	2.01	1.91	1.81b	.050	2.03a	1.86	1.94
R5g_Tobacco	.572	1.87	1.87	1.75	1.79	1.75	.531	1.77	1.80	0.007	1.89a	1.77	1.70	.000	1.91a	1.77	1.67	.013	1.95a	1.77	1.73
R5h_Pharma	.031	2.16a	1.94	1.89	2.07	2.03	.803	2.01	1.99	0.282	2.01	2.05	1.94	.005	2.09	2.03	1.88b	.301	2.09	1.97	2.01
R5i_Insurance	<.000	2.63a	2.08	2.04	2.09	2.02	.651	2.09	2.12	<.000	2.08c	2.32c	1.92c	.000	2.26a	2.10	1.96	<.000	2.42c	2.11c	1.94c
**WPRDB Total Score**	<.000	2.67a	2.43	2.31b	2.48	2.55	.594	2.45	2.47	0.001	2.40	2.56a	2.41	.836	2.44	2.46	2.47	.352	2.47	2.43	2.49

* CHL scores range 0 to 30: Higher numbers indicate higher level of cancer health literacy
** WPRDB scores range 1 to 4: Higher numbers indicate higher level of willingness to participate
(a) These groups had significant HIGHER mean scores than some of their counterparts (p<0.5).
(b) These groups had significant LOWER mean scores than some of their counterparts (p<0.5).
(c) These groups were significantly different among the other groups (p<0.5).
